# Effects of Sildenafil on Cognitive Function Recovery and Neuronal Cell Death Protection after Transient Global Cerebral Ischemia in Gerbils

**DOI:** 10.3390/biomedicines12092077

**Published:** 2024-09-12

**Authors:** Yeon Hee Yu, Gun Woo Kim, Yu Ran Lee, Dae-Kyoon Park, Beomjong Song, Duk-Soo Kim

**Affiliations:** 1 Department of Anatomy, College of Medicine, Soonchunhyang University, Cheonan 31151, Republic of Korea; yyh0220@sch.ac.kr (Y.H.Y.); kbk1999@sch.ac.kr (Y.R.L.); mdeornfl@sch.ac.kr (D.-K.P.); 2 Research Supporting Center for Medical Science, College of Medicine, Dong-A, Busan 49201, Republic of Korea; yh03190@dau.ac.kr

**Keywords:** global ischemia, reperfusion, sildenafil, behavioral test, neuroprotection

## Abstract

Cerebral ischemic stroke is a major cause of death worldwide due to brain cell death resulting from ischemia-reperfusion injury. However, effective treatment approaches for patients with ischemic stroke are still lacking in clinical practice. This study investigated the potential neuroprotective effects of sildenafil, a phosphodiesterase-5 inhibitor, in a gerbil model of global brain ischemia. We investigated the effects of sildenafil on the expression of glial fibrillary acidic protein and aquaporin-4, which are markers related to astrocyte activation and water homeostasis, respectively. Immunofluorescence analysis showed that the number of cells co-expressing these markers, which was elevated in the ischemia-induced group, was significantly reduced in the sildenafil-treated groups. This suggests that sildenafil may have a potential mitigating effect on astrocyte activation induced by ischemia. Additionally, we performed various behavioral tests, including the open-field test, novel object recognition, Barnes maze, Y-maze, and passive avoidance tests, to evaluate sildenafil’s effect on cognitive function impaired by ischemia. Overall, the results suggest that sildenafil may serve as a neuroprotective agent, potentially alleviating delayed neuronal cell death and improving cognitive function impaired by ischemia.

## 1. Introduction

Stroke is a major cause of morbidity and mortality worldwide [1]. According to the WHO, the global prevalence of stroke is approximately 0.08%, with 15 million people suffering from stroke worldwide, of whom about 5 million experience permanent disabilities [2,3]. Strokes are classified into two types: ischemic and hemorrhagic, with ischemic stroke accounting for 50–85% of strokes [2,3]. Ischemic stroke, resulting from disrupted blood flow to the brain, causes brain cell death and diverse impairments, including physical, cognitive, and emotional challenges, and leads to death in severe cases [4,5,6,7,8,9,10,11]. Although numerous treatment methods for stroke have been suggested, they are not very effective in actual clinical practice, contrary to the promising results from preclinical studies [7,12,13,14]. Therefore, the only available treatment for ischemic stroke aims only to restore normal blood flow and involves the administration of thrombolytic agents [15]. However, this treatment is limited in that it should be administered within 4.5 h after the onset of cerebral ischemia [16].

Sildenafil, a type of phosphodiesterase-5 (PDE5) inhibitor, was initially developed and approved as a medication for the treatment of erectile dysfunction in men. The inhibition of PDE5 by sildenafil increases the intracellular level of cyclic guanosine monophosphate (cGMP), which, in turn, leads to vasodilation and increases in blood flow [17,18,19]. The increased blood flow by sildenafil is effective for improving symptoms such as pulmonary hypertension, heart failure, inflammatory response, and fibrin deposition [19,20,21]. In particular, sildenafil has been effective in various animal models of brain disorders by providing neuroprotection, restoring neuronal development, improving brain injury, and enhancing cognitive function [22,23,24]. However, the effects of sildenafil on delayed neuronal cell death and cognitive impairment following global ischemia, as well as its therapeutic mechanisms, remain unknown.

Therefore, in this study, we evaluated the therapeutic effects of sildenafil on transient global ischemia. The use of sildenafil for the treatment of global ischemia has not been extensively studied, and this research will provide valuable information regarding its potential effects.

## 2. Materials and Methods

### 2.1. Experimental Animals

Mongolian gerbils (Meriones unguiculatus) weighing 60–80 g were used for all experiments. The animals were obtained from the Animal Experiment Center at Soonchunhyang University in Cheonan, Republic of Korea. The gerbils were housed under controlled conditions, with temperature maintained at 22 ± 2 °C, humidity at 55 ± 5%, and a 12:12 light/dark cycle. They had ad libitum access to a commercial diet and water. All animal protocols were approved by the Soonchunhyang University Animal Experimentation Panel (date of approval: 20 April 2022, permit No. SCH22-0086). All efforts were made to minimize the number of animals used and their suffering.

### 2.2. Induction of Transient Global Cerebral Ischemia

The Mongolian gerbil, with an approximately 70% incomplete circle of Willis, is a useful animal model for studying the mechanisms of delayed neuronal death following global cerebral ischemia [25,26]. Therefore, it serves as a good screening model for evaluating neuroprotective drugs related to brain ischemia [27]. The gerbils were anesthetized with a mixture of 33% oxygen and 67% nitrous oxide containing isoflurane. Both common carotid arteries were isolated and occluded using atraumatic aneurysm clips. Complete occlusion was confirmed by visualizing the common carotid artery using an ophthalmoscope. The aneurysmal clips were removed after a 5-min occlusion of the common carotid artery. Body (rectal) temperature was monitored using a rectal temperature probe and maintained at 36.7–37 °C with a heating pad equipped with a thermostat.

### 2.3. Drug Administration

The efficacy of sildenafil was evaluated by intraperitoneal injection of sildenafil citrate (SIL, 10 mg/kg or 20 mg/kg; Hanmi Parm. Co., Seoul, Republic of Korea) dissolved in 0.8% saline solution 30 min before and after the induction of transient global cerebral ischemia [24,28]. To compare the sildenafil dosage used in the ischemia experiments with the human dosage, we calculated the human equivalent dose (HED) using the body surface area-based conversion method and the equation below [29].

HED (mg/kg) = animal does (mg/kg) × (animal K_m_/human K_m_)

Using the above equation to calculate the HED, the HED for the 10 or 20 mg/kg doses administered in the gerbil experiment is approximately 1.35 or 2.7 mg/kg. The clinical dose of sildenafil corresponds to 50–100 mg per single intake when calculated for an adult weighing 60 kg. Hence, the 10 mg/kg dose of sildenafil used for gerbil ischemia falls within the permissible range, whereas the 20 mg/kg dose exceeds the allowable dose by more than twice. We divided the gerbils into 6 groups as follows: Group 1, control; Group 2, ischemic; Group 3, SIL-pre (10 mg/kg); Group 4, SIL-post (10 mg/kg); Group 5, SIL-pre (20 mg/kg); and group 6, SIL-post (20 mg/kg). In behavioral studies, we typically used more than 10 animals per group to achieve non-parametric statistical analysis with sufficient power [30].

### 2.4. Behavioral Tests

**Figure 1:**
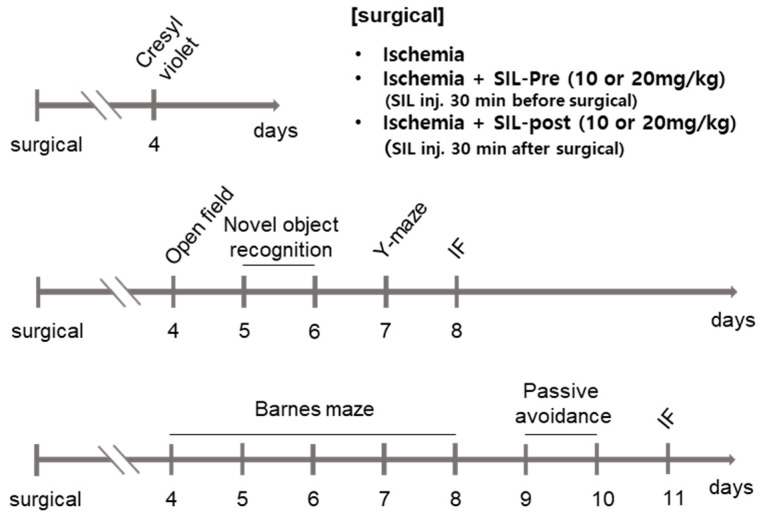
A representative diagram shows the overall surgical groups, behavior tests, and histology time schedules. Cresyl violet staining was performed on the fourth day after surgery. For the behavioral analysis, to prevent learning of the special cue, the Barnes maze and Y-maze tests were not performed on the same gerbils. Additionally, the behavioral analysis was conducted in two parts to minimize stress on the animals for each task. After completing the behavioral task, we collected tissue samples and performed immunofluorescence staining. SIL, sildenafil; IF, Immunofluorescence.

The animals were exposed to white noise consisting of a combination of 60 Hz frequency sounds for 1 h before the behavioral tests to facilitate adaptation to various behavioral test environments. All experimental animals underwent behavioral testing 4 days after ischemia. The behavioral tests were recorded and analyzed using a PC-based video behavioral analysis system, EthoVision XT 14 (Noldus, Wageningen, The Netherlands), with automated tracking software. We conducted cognitive-related behavioral tests and performed tissue staining on six groups of gerbils using the procedures described in Figure 1.

#### 2.4.1. Open-Field Test

The open-field test (OFT) assesses general motor activity and anxiety. The maze arena consists of a 40 × 40 × 40 cm open-field apparatus with diffused lighting. The maze is virtually divided into a central zone and a peripheral zone. The gerbils were placed in the center of the zone and allowed to explore freely for 30 min. The total distance moved determined locomotor activity, and the time spent in the central zone was evaluated as the index of anxiety [31,32].

#### 2.4.2. Novel Object Recognition

Object exploratory behavior was investigated using a novel object to exploit the basic exploration characteristics of rodent species. The induction and maintenance of activity vary depending on the difference in response (recognition) to a novel physical object in a familiar place. The novel object recognition (NOR) test used in the OFT in this study was a white acrylic box. The animals were familiarized with the box for 30 min a day before the experiment. In the habituation phase the next day, all groups were placed in the same chamber with 2 similar objects and freely explored them for 10 min before returning to their cage. In the test phase 24 h later, one of the objects was replaced with a new object with a different shape and color. Animals were placed back in the test chamber with the novel and previously explored (familiar) objects for 10 min. At each phase, the animals were placed in the center of the box, and their gaze was directed toward the opposite wall of the two objects. Exploration behavior was analyzed through various actions, such as approaching, smelling, licking, and direct contact with the forefoot. We evaluated the ability of the animals to remember familiar objects. Object exploration was defined as the time spent by animals in direct contact with the object (e.g., smelling the object or being less than 1 cm from the object) [33].

#### 2.4.3. Barnes Maze

The Barnes maze consists of a circular platform (92 cm in diameter) with 20 holes evenly spaced around the circumference and located 105 cm above the floor. Four different visual signals were placed on the walls surrounding the platform in each quadrant for the animals to use as cues. For safety, an escape chamber was located under one of the holes (escape hole). Bedding was added to the chamber to allow the animals to hide. A metronome (80 Hz noise) and high lighting (300 lux lighting at the center of the platform) were used to amplify anxiety and motivate the animals to find the escape hole. The gerbils interacted with the Barnes maze in 3 stages: habituation (1 day), training (2−4 days in a long-term training paradigm), and probe (5 days). During the four-day adaptation phase, animals were allowed to explore the platform from the center when a black cylinder was removed 10 s later. Their activities were recorded with a video camera. If the animals found the escape hole within 180 s, the hole was covered with a lid for 120 s to block light and stop electronic sounds. Otherwise, animals were carefully guided to the hole with the escape chamber to induce learning of the space. Each animal was tested 3 times at 15-min intervals in the acquisition-training phase to improve spatial memory for finding the escape hole, and the time it took to find the escape hole on the fifth day was measured as the spatial acquisition phase (probe phase). The escape chamber was removed, and this stage was performed only once, unlike the 3 times in the adaptation stage [34,35].

#### 2.4.4. Y-Maze

Spontaneous alternation was assessed in the Y-maze test as previously described [33]. The Y-maze consisted of 3 arms (40 × 5 × 20 cm; length, width, height) marked A, B, and C, which were equidistant and converged at a 120° angle. Each animal was placed at the end of one arm and allowed to freely explore for 7 min. The order (e.g., ABC, BCA, and CAB) and the number of arm entries were documented manually for each animal during the 7-min trial. Spontaneous alternation behavior was defined as consecutive selections of ABC, CAB, or BCA without choosing ABA and entering all 3 arms. The maze was thoroughly cleaned with 70% ethanol and distilled water between trials to eliminate residual odor. Spontaneous alternation behavior was determined using the equation below:


% alternation = [(number of alternations)/(total arm entries − 2)] × 100

The Y-maze, used to assess hippocampal-dependent spatial working memory, was placed in a secluded room with specific cues on each arm. The remaining recording and analysis were conducted in the same manner as described above.

#### 2.4.5. Passive Avoidance

The passive avoidance test was conducted using 2 chambers of equal size, one brightly lit and the other dark, separated by a door with an electric grid floor that allowed the electric current to pass through. During the acquisition trial phase, a gerbil was initially placed and allowed to explore the bright chamber for approximately 120 s. When the door to the dark chamber was opened, the animal moved, causing the door to close automatically. Training trials were conducted for 24 h after the acquisition attempts. The gerbil was allowed to explore the bright chamber for about 120 s, after which the door was opened. As soon as the gerbil entered the dark chamber, the door closed automatically, and an electric shock (1 mA for 2 s) was delivered through the grid floor. After 24 h of training, the testing trial was performed. The retention time was estimated by measuring the time taken by the gerbil to enter the dark chamber after opening the door within a 180-s time. If the gerbil did not enter the dark chamber within 180 s, a retention time of 180 s was recorded [36].

### 2.5. Tissue Processing and Cresyl Violet Staining

Anesthetized animals (with urethane 1.5 g/kg) were perfused with saline via the heart, followed by an injection of 4% paraformaldehyde in 0.1 M phosphate buffer (PB). The brain was removed and post-fixed in the same fixative for 4 h, then exchanged with 30% sucrose with PB for 2 days at 4 °C. The tissue was frozen and sectioned at 30 µm thickness using a cryostat and collected onto 6-well plates containing phosphate-buffered saline (PBS) storage solution. Cresyl violet staining was used to investigate the effects of ischemia on cell death or damage, following the procedure described previously [24]. Tissue sections were mounted onto gelatin-coated microscope slides and stained with cresyl violet acetate (Sigma, St. Louis, MO, USA) for 20 min at room temperature. The slides were then washed with distilled water, dehydrated in 50%, 70%, 80%, 90%, 95%, and 100% ethanol sequentially for 5 min each at room temperature, and finally mounted with Canada balsam (Kanto, Tokyo, Japan). After that, images were captured for cell death or damage following ischemia measurements using an Axiocam digital camera and ZEN 3.4 microscope digital camera software (Zeiss, Aalen, Germany).

### 2.6. Double Immunofluorescence

Double-immunofluorescence staining for aquaporin-4 (AQP-4) and glial fibrillary acidic protein (GFAP) was performed to assess morphological changes in the blood–brain barrier (BBB) induced by ischemia-reperfusion in the same hippocampal tissue and changes induced by sildenafil. Brain tissues were incubated overnight at 4 °C in a mixture containing primary antibodies in PBS with 0.3% Triton X-100 for 48 h: mouse anti-GFAP IgG (Millipore, Burlington, MA, USA; diluted 1:500). After the sections were incubated at room temperature for 3 h with Cy2-conjugated secondary antibody, diluted 1:200 (Jackson Immuno Research Labs, West Grove, PA, USA). Following this, the same brain tissues were incubated with a different set of primary antibodies, rabbit anti-AQP-4 IgG (Alomone Labs, Jerusalem, Israel; diluted 1:200), for 48 h at 4 °C. Subsequently, the tissues were incubated with a Cy3-conjugated secondary antibody for 3 h at room temperature. After PBS wash, the tissues were incubated with DAPI (Invitrogen, Waltham, MA, USA) diluted 1:500 for 10 min at room temperature. The slices were mounted on slides and cover-slipped with DPX (Sigma, St. Louis, MO, USA). All images were captured using a model Fluoview FV10i microscope and FV10i software (Olympus, Tokyo, Japan).

### 2.7. Quantification of Data and Statistical Analysis

Data quantification and statistical analysis were conducted as described in the previous study, with some modifications [37]. Cell numbers were estimated using optical fractionation, a stereological technique that combines optical dissection counting with fractionator sampling. This method relies on a well-designed, systematic random sampling approach. The approach, by definition, provides unbiased estimates of a population. Deep tissue samples were used, with an optical dissector height (h) of 30 µm. All data were analyzed using GraphPad Prism 8.0.1 (GraphPad Software, San Diego, CA, USA) and are presented as the mean ± standard deviation. One-way analysis of variance (ANOVA), followed by Dunnett’s multiple comparison test. A pairwise comparison was conducted to compare the ischemia gerbils to control gerbils, as well as compare the sildenafl-treated gerbils to ischemia gerbils [38,39]. Statistical significance was defined as * *p* < 0.05, ** *p* < 0.01, and *** *p* < 0.001.

## 3. Results

### 3.1. Neuroprotective Effects of Sildenafil on Neuronal Cell Death Following Transient Global Cerebral Ischemia-Reperfusion

**Figure 2:**
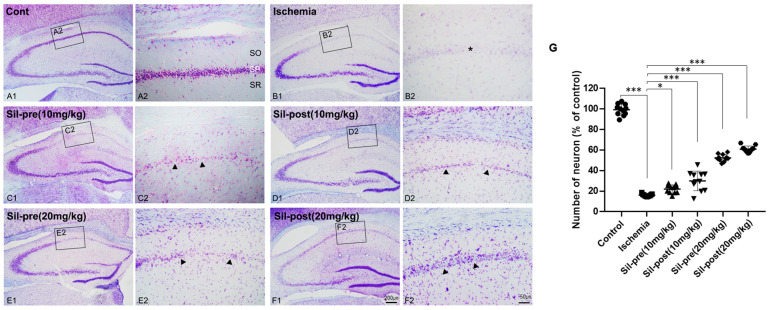
Sildenafil treatment increased cell populations in the cornu ammonis 1 (CA1) region compared to transient global cerebral ischemia. In the cresyl violet staining, the number of neurons in the stratum pyramidale (SP, asterisk) of the CA1 field was reduced in ischemic gerbils compared to control gerbils (**A1**–**B2**,**G**). S74IL-pre (10 mg/kg) gerbils showed slight recovery (**C1**,**C2**,**G**). The number of neurons in SIL-post (10 mg/kg) gerbils was significantly increased compared to ischemic gerbils (**D1**,**D2**,**G**, arrowhead). The number of neurons in the SP area in the SIL-treated (20 mg/kg) groups was significantly increased compared to the ischemic group (**E1**–**F2**,**G**, arrowhead). SO, stratum oriens; SP, stratum pyramidale; SR, stratum radiatum. Scale bar = panels **A1**–**F1**, 200 μm; panels **A2**–**F2**, 50 μm. Data are presented as means ± standard errors of the mean. * *p* < 0.05, *** *p* < 0.001 by one-way analysis of variance (ANOVA) followed by Dunnett’s multiple comparisons test, *n* = 11/group.

First, we tested the neuroprotective effects of sildenafil on brains with ischemia. Cresyl violet staining was performed to assess neuronal cell death and damage in the hippocampus of the animal model after transient global cerebral ischemia-reperfusion (Figure 2). Responses to Nissl bodies in the control gerbils were predominantly detected in hippocampal cornu ammonis 1 (CA1) neurons and various neuronal cell bodies, encompassing the majority of the neuronal cell populations (Figure 2A1,A2). Most neuronal populations in the hippocampal CA1 region of ischemic animals showed significant cell death (Figure 2B1,B2). Quantitative analysis of viable neurons yielded results consistent with the histological examination of cresyl violet staining (ischemia group, *p* < 0.001; Figure 2G). Sildenafil pre-treated (SIL-pre 10 mg/kg) gerbils showed changes in neuroprotection (*p* = 0.034; Figure 2C1,C2,G). In contrast, the number of neurons in the hippocampal CA1 region increased in SIL-post (10 mg/kg) gerbils compared to ischemic gerbils (*p* < 0.001; Figure 2D1,D2,G). Both the SIL-pre and SIL-post (20 mg/kg) gerbils showed significant increases in the expression of Nissl bodies compared to ischemic gerbils (*p* < 0.001; Figure 2E1–F2,G).

### 3.2. Effects of Sildenafil on AQP4 and GFAP Expression Following Transient Global Cerebral Ischemia-Reperfusion

**Figure 3:**
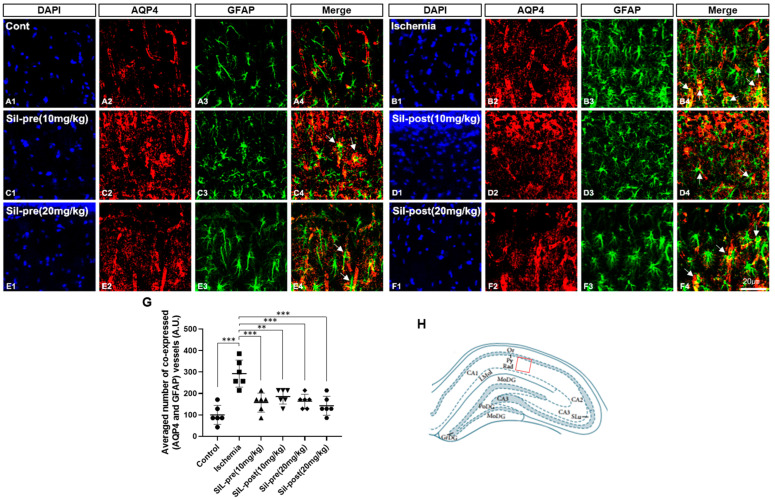
Sildenafil treatment decreased aquaporin-4 (AQP4) and glial fibrillary acidic protein (GFAP) co-expression in the CA1 region compared to transient global cerebral ischemia. The co-expression of AQP4 and GFAP was significantly increased in ischemic gerbils compared to control gerbils (**A1**–**B4**,**G**, arrow). Co-expression was significantly decreased in sildenafil-treated gerbils compared to ischemic gerbils (**C1**–**F4**,**G**, arrow). Scale bar = 20 μm. Diagram of the gerbil hippocampus and the red square indicates the area where IF imaging was performed (**H**). Data are presented as means ± standard errors of the mean. ** *p* < 0.01, *** *p* < 0.001 by one-way ANOVA followed by Dunnett’s multiple comparisons test, *n* = 11/group.

Double-staining for AQP4 and GFAP was performed to assess the functional changes induced by transient cerebral ischemia-reperfusion and sildenafil treatment in the hippocampus by BBB disruption (Figure 3). GFAP and AQP4-positive immunoreactivity were significantly increased in the hippocampal CA1 region of the ischemic gerbils compared to control gerbils (Figure 3A1–A3,B1–B3). The number of double-labeled astrocytes and vessels, which express both AQP4 and GFAP, in the hippocampal CA1 region was increased in ischemic gerbils compared to control gerbils (*p* < 0.001; Figure 3A4,B4,G). In contrast, in all of the sildenafil pre- and post-treatment groups, the double-labeling of AQP4 and GFAP was significantly restored to levels comparable to control gerbils (SIL-pre and -post (10 mg/kg), *p* < 0.001 and *p* < 0.01; SIL-pre and -post (20 mg/kg), *p* < 0.001; Figure 3C4–F4,G). AQP4 immunoreactivity was not decreased in any group (Figure 3A2–F2), whereas GFAP immunoreactivity was decreased in all groups administered sildenafil compared to ischemic gerbils (Figure 3B3–F3).

### 3.3. Hyper-Locomotion Activity Following isChemia in Gerbils Treated with Sildenafil

**Figure 4:**
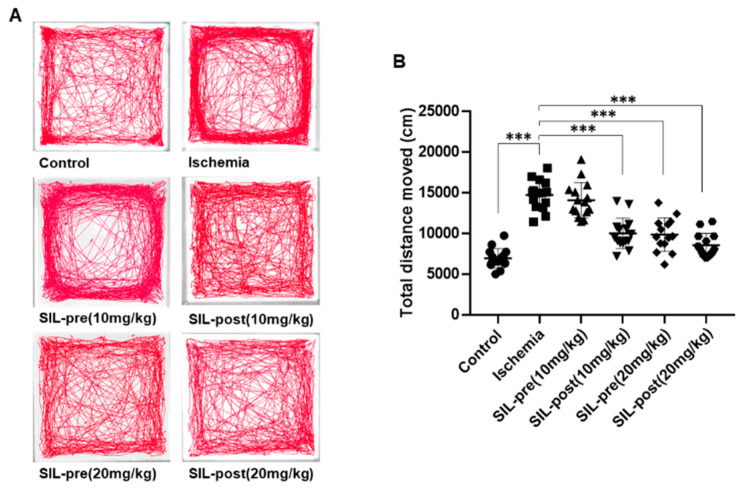
Sildenafil treatment restored generalized locomotor activity in transient global cerebral ischemia. Representative cumulative traces of navigation pathways in the control, ischemic, SIL-pre, and SIL-post groups during exploration in the open-field test (**A**). The total distance moved by ischemic gerbils was increased compared to control gerbils (**B**). The total distance moved by sildenafil-treated gerbils was significantly reduced compared with ischemic gerbils, except for SIL-pre (10 mg/kg) gerbils (**B**). Data are presented as means ± standard errors of the mean. *** *p* < 0.001 by one-way ANOVA followed by Dunnett’s multiple comparisons test, *n* = 15/group.

The OFT was conducted on day 4 after ischemia-reperfusion to assess the changes in general locomotor activity observed in experimental animals after ischemic stroke and evaluate the impact of sildenafil (Figure 4). Representative tracings of each group are displayed in Figure 4A, and the movement tracks of each group (line) are indicated. The ischemic gerbils showed a significant increase in the total distance moved compared to control gerbils (*p* < 0.001; Figure 4A,B). No change in the total distance moved was seen in SIL-pre (10 mg/kg) gerbils, whereas a significant decrease was observed in SIL-post (10 mg/kg) gerbils compared to ischemic ones (SIL-pre (10 mg/kg), *p* = 0.785; SIL-post (10 mg/kg), *p* < 0.001; Figure 4A,B). In contrast, both the SIL-pre and SIL-post (20 mg/kg) gerbil groups exhibited a decrease in the total distance moved to the level of the control gerbils (SIL-pre (20 mg/kg), *p* < 0.001; SIL-post (20 mg/kg), *p* < 0.001; Figure 4A,B).

### 3.4. Sildenafil Treatment Alleviates Deficits in Cognitive Behavior Following Ischemia in Gerbils

**Figure 5:**
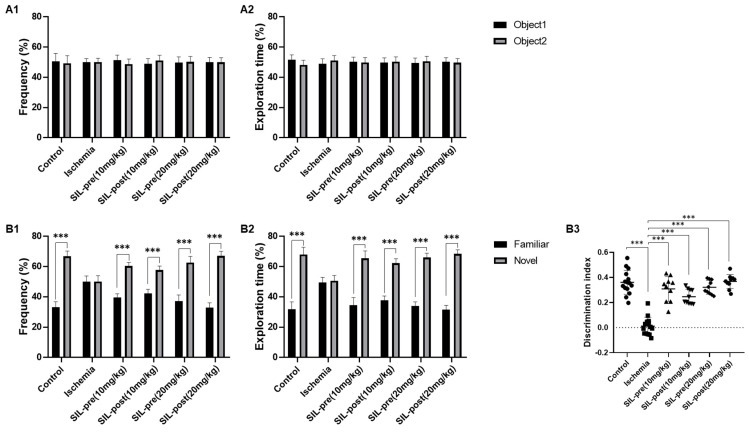
Sildenafil treatment alleviated novel object recognition deficits induced by transient global cerebral ischemia. In the habituation phase, the frequency percentage of searching for the same two objects was similar in the control, ischemic, and sildenafil-treated groups (**A1**). The percentage of time spent on object exploration was similar in the control, ischemic, and sildenafil-treated groups (**A2**). In the test phase, the frequency and percentage of time spent exploring novel objects were decreased in ischemic gerbils compared with control gerbils (**B1**,**B2**). However, both the frequency and percentage of time spent exploring novel objects in sildenafil-treated gerbils were significantly increased as compared to ischemic gerbils (**B1**,**B2**). In particular, the discrimination index of the sildenafil-treated groups was notably different compared with the ischemic group (**B3**). Data are presented as means ± standard errors of the mean. *** *p* < 0.001 by one-way ANOVA followed by Dunnett’s multiple comparisons test, control, and ischemia, *n* = 11; SIL-groups, *n* = 15.

The NOR test was performed to evaluate cognitive function related to the exploration of novel objects [33]. During the habituation phase, no statistically significant differences were seen in exploration frequency or time percentage between gerbils in the control, ischemic, and sildenafil groups, indicating comparable adaptation patterns between two identical objects (Figure 5A1,A2). In the test phase, the control gerbils showed a significant increase in exploration frequency and time percentage for the novel object compared to the familiar object (control group, frequency and exploration time, *p* < 0.001; Figure 5B1,B2). However, in the ischemic gerbils, no statistical differences were found in these measures, indicating similar preferences for the novel and familiar objects (ischemic group, frequency, *p* = 0.999; exploration time, *p* = 0.9368 by one-way ANOVA; Figure 5B1,B2). The frequency and exploration time percentage for the novel object were significantly increased in all sildenafil-treat gerbils, similar to the control gerbils (SIL-pre and SIL-post (10 mg/kg), frequency and exploration time, *p* < 0.001; SIL-pre and SIL-post (20 mg/kg), frequency and exploration time, *p* < 0.001 by one-way ANOVA; Figure 5B1,B2). In addition, the discrimination index between familiar and novel objects was reduced in ischemic gerbils but significantly improved in all sildenafil-treated animals (ischemic group, *p* < 0.001; SIL-pre and SIL-post (10 mg/kg), *p* < 0.001; SIL-pre and SIL-post (20 mg/kg), *p* < 0.001 by one-way ANOVA; Figure 5B3).

**Figure 6:**
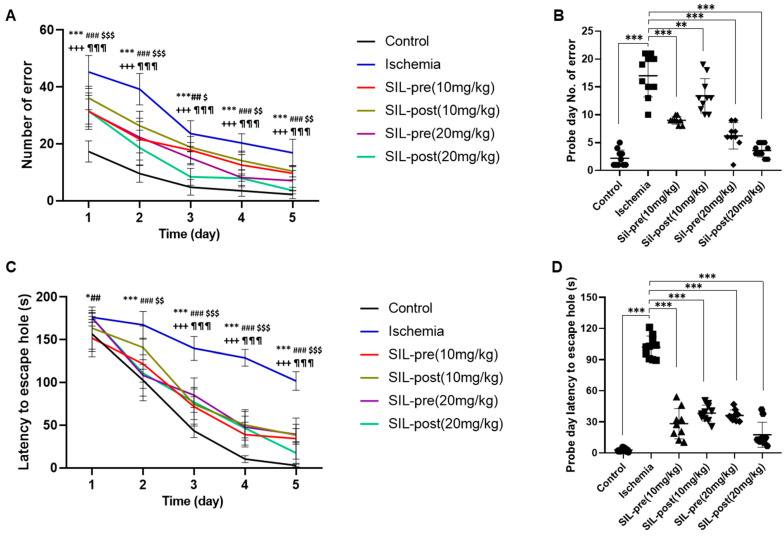
Sildenafil treatment improved long-term memory, spatial learning, and memory impairment caused by transient global cerebral ischemia. In the Barnes maze test conducted for 5 days, the number of errors in all groups decreased (**A**). On the probe day, the number of errors made by ischemic gerbils increased significantly compared to control gerbils (**B**). The number of errors was significantly reduced on the probe test day in all sildenafil-treated groups as compared to ischemic gerbils (**A**,**B**). The latency time to the escape hole decreased in all groups over 5 days (**C**). On the probe day, the latency time to the escape hole was significantly increased in ischemic gerbils compared to control gerbils (**D**). In all sildenafil-treated groups, the latency time to the escape hole was significantly reduced compared to the ischemic group (**C**,**D**). Data are presented as means ± standard errors of the mean. * *p* < 0.05, *** *p* < 0.001 control group vs. ischemic group; ^##^ *p* < 0.01, ^###^ *p* < 0.001 ischemic group vs. SIL-pre (10 mg/kg); ^$^ *p* < 0.05, ^$$^ *p* < 0.01, ^$$$^ *p* < 0.001 ischemic group vs. SIL-post (10 mg/kg); ^+++^ *p* < 0.001 ischemic group vs. SIL-pre (20 mg/kg); ^¶¶¶^ *p* < 0.001 ischemic group vs. SIL-post (20 mg/kg) (**A**,**C**). ** *p* < 0.01, *** *p* < 0.001 (**B**,**D**) by one-way ANOVA followed by Dunnett’s multiple comparisons test, *n* = 10/group.

The Barnes maze test was performed to investigate spatial reference learning and memory (Figure 6). All groups showed improvements throughout the training phase in the number of errors and latency time in finding the escape hole through repeated attempts (Figure 6A,C). In the probe phase, the number of errors and latency time to the escape hole were significantly increased in ischemic gerbils compared to control gerbils (ischemic group, number of errors and latency, *p* < 0.001; Figure 6B,D), indicating a pronounced learning deficit during this period. In contrast, regardless of the dosage, all sildenafil-treated groups showed a significant reduction in the number of errors (SIL-pre (10 mg/kg), *p* < 0.001; SIL-post (10 mg/kg), *p* < 0.01; SIL-pre (20 mg/kg) and SIL-post (20 mg/kg), *p* < 0.001; Figure 6B) and in latency time to the escape hole on the probe test day compared with ischemic gerbils (SIL-pre and SIL-post (10 mg/kg), *p* < 0.001; SIL-pre and SIL-post (20 mg/kg), *p* < 0.001; Figure 6D).

**Figure 7:**
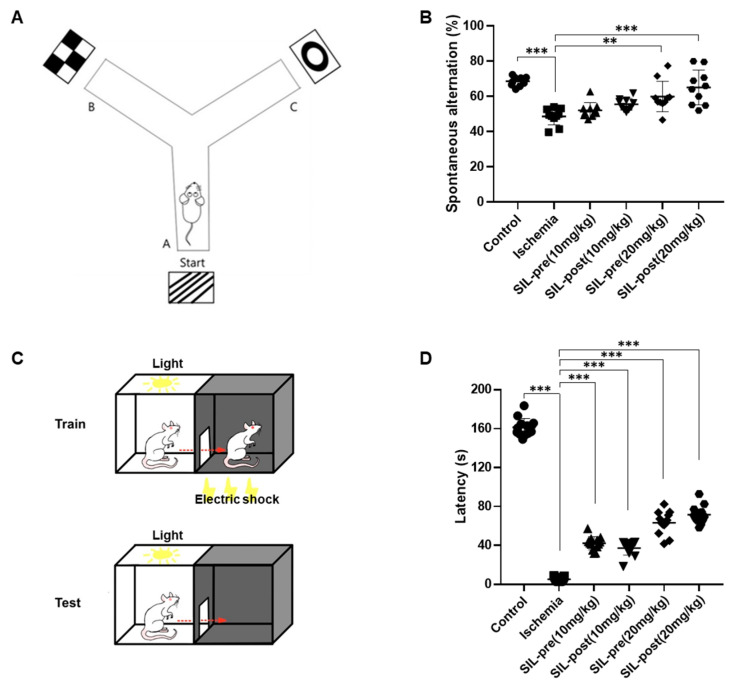
Sildenafil treatment alleviated the spatial and emotional memory deficits induced by transient global cerebral ischemia. A schematic diagram showing special cues in the working memory test (**A**). The average percentage of spontaneous alternations in ischemic gerbils was noticeably reduced compared with control gerbils (**B**). However, spontaneous alternations were significantly increased in the sildenafil-treated (20 mg/kg) groups compared to ischemic gerbils (**B**). A schematic diagram showing the passive avoidance test (**C**). The transfer time was significantly decreased in the ischemic group compared to the control group (**D**). In contrast, the latency time in all sildenafil-treated groups was increased compared to the ischemic group (**D**). Data are presented as means ± standard errors of the mean. ** *p* < 0.01, *** *p* < 0.001 ANOVA followed by Dunnett’s multiple comparisons test, Y-maze, *n* = 10/group; PA, *n* = 13/group.

The Y-maze task was utilized to evaluate hippocampus-dependent spatial working memory, which is a form of short-term memory, and the passive avoidance test was used to evaluate emotional short-term memory (Figure 7A,C) [33,36]. The average percentage of spontaneous alternations in ischemic gerbils was significantly decreased compared to control gerbils (*p* < 0.001; Figure 7B), indicating impaired memory and spatial working memory. Both sildenafil-treated (10 mg/kg) gerbils showed a tendency toward increased voluntary spontaneous alternations. However, this difference was not statistically significant compared to ischemic gerbils (SIL-pre (10 mg/kg), *p* = 0.5873; SIL-post (10 mg/kg), *p* = 0.0611; Figure 7B). However, both the SIL-pre and SIL-post treated (20 mg/kg) groups showed an increase in the average percentage of spontaneous alternations compared to the ischemic group (SIL-pre (20 mg/kg) and SIL-post (20 mg/kg), *p* < 0.001; Figure 7B). The experimental results for passive avoidance indicated a significant decrease in latency in the ischemic group compared to the control group (*p* < 0.001 by one-way ANOVA; Figure 7D). An increase in latency was seen in all sildenafil-treated gerbils compared to ischemic gerbils (SIL-pre and SIL-post (10 mg/kg), *p* < 0.001; SIL-pre and SIL-post (20 mg/kg), *p* < 0.001; Figure 7D). As the sildenafil dosage increased, there was a tendency for transfer latency to increase. These results suggest that sildenafil may improve cognitive function in brains affected by ischemic damage.

## 4. Discussion

In this study, we confirmed the beneficial efficacy of sildenafil treatment in improving after-stroke symptoms. Sildenafil plays a crucial role in stabilizing cGMP, thereby acting as an important regulator of nitric oxide (NO) signaling, which increases cerebral blood flow and enhances neuroprotective effects [40]. Brain edema is a serious complication of stroke, and previous studies reported that cellular edema in ischemia primarily affects astroglia, increasing glial K^+^ uptake [41]. The induction of long-term potentiation (LTP), associated with neuronal excitation, is also affected by changes in extracellular potassium [42]. Previous studies have found that in AQP4-deficient mice, K^+^ reuptake is slowed. This reduction in potassium reuptake leads to an increase in extracellular potassium, resulting in the depolarization of both neurons and glial cells [43,44]. In our current results, the reduction in edema formation due to the attenuation of astrocyte reactivity following sildenafil treatment in ischemia gerbils may increase extracellular potassium, causing tonic depolarization of neurons. This could lead to greater postsynaptic depolarization, thereby improving LTP [44]. Additionally, AQP4-knockout mice exhibited neuroprotection and improved inflammation due to reductions in brain edema in transient cerebral ischemia [41,45]. Sildenafil inhibits microglia activation and the death of oligodendrocytes through the mitogen-activated protein kinase (MAPK) signaling pathway, while increases in the production of pro-inflammatory cytokines via protein kinase G (PKG) signaling affects the activation of astrocytes and microglia [23,46,47,48]. The double-immunofluorescence analysis of GFAP and AQP-4 co-expression provides evidence of astrocytic activation and water regulatory mechanisms in response to global ischemic insults. Our research findings suggest that downregulation of GFAP and AQP-4 co-expression after sildenafil administration potentially mitigates astrocytic reactivity and reduces edema formation, supporting previous studies demonstrating sildenafil’s anti-inflammatory and neuroprotective properties [24]. These findings contribute to our understanding of the complex cellular interactions involved in ischemic events and highlight the therapeutic potential of sildenafil in mitigating astrocyte-mediated damage.

Previous research reported that gerbils exhibited hyperactivity in the early stages following ischemia [27,49,50] and suggested that cell loss induced by global ischemia, along with the consequent loss of spatial mapping function, contributes to hyperlocomotion in the OFT [27]. Improvements in hippocampal delayed neuronal death by neuroprotective agents has been claimed to ameliorate hyperactivity, suggesting a close association between hyperlocomotion and hippocampal delayed neuronal death [27]. Consistent with these findings, our results also observed a reduction in cell death through cresyl staining and decreased hyperactivity in the OFT following sildenafil treatment. Further investigations are needed to clarify the causal relationship of these changes. 

We utilized the NOR test, Barnes maze, Y-maze with a special cue, and the passive avoidance test to assess hippocampal-dependent memory [33,37,51,52,53,54,55]. The Barnes maze test serves as a dry-land-based test for spatial learning and memory [53,54]. It represents a hippocampus-dependent task where animals learn the relationship between distal cues in the surrounding environment and a fixed escape location [54]. Spontaneous alternation behavior in the Y-maze test is commonly interpreted as a task evaluating working memory in a novel environment [51,52]. We conducted Y-maze tests using spatial cues to evaluate hippocampus-dependent spatial working memory [37]. The passive avoidance test was employed as a measure of aversive memory [45]. It is recognized for its ability to assess an animal’s ability to remember and avoid unpleasant stimuli [56,57]. In our study, ischemic gerbils exhibited impairments in spatial learning and cognitive function similar to previous research findings, aligning with existing knowledge that highlights the vulnerability of hippocampal-dependent tasks to ischemic damage [33,58,59]. In the present results, the cognitive impairment of novel objects in the ischemia group increased to the level of the control group regardless of the dose and timing of sildenafil administration (Figure 4). The group treated with sildenafil showed significant improvement in spatial learning, spatial memory, working memory, and aversive memory, indicating a potential ameliorative effect of sildenafil not only on spatial learning and memory but also on working and aversive memory following an ischemic insult. However, the current results show that the improvement in aversive memory did not recover to control levels compared to other cognitive function analyses. This indicates the need for further detailed studies on the amygdala neural circuitry to understand sildenafil’s effect on improving aversive memory [60]. The inhibition of the PDE5 effect by sildenafil increases intracellular cGMP levels by breaking down the phosphodiester bond of cGMP and inhibiting the hydrolysis of cGMP to GMP [23]. Previous studies have proposed that sildenafil activates the PI3K/Akt pathway via the NO-cGMP-PKG signaling pathway, enhancing neurogenesis and synaptic plasticity [61,62]. Consequently, sildenafil reduces neuronal loss and protects the structure of synapses, effectively defending the neural network [63,64]. Astrocytic processes contact multiple neuronal membranes, forming tripartite synapses that enclose pre- and postsynaptic elements, thereby establishing close contacts between neurons [65,66]. During this process, astrocytes secrete gliotransmitters such as glutamate, ATP, and D-serine, directly influencing neuronal functions and playing a crucial role in synaptic plasticity [66,67,68,69]. In AQP4-deficient mice, reduced levels of glutamate transporter-1 lead to excessive accumulation of glutamate, which significantly increases N-methyl-D-aspartate receptor (NMDAR)-mediated currents [42,70,71]. Since the postsynaptic activation of NMDARs is essential for LTP induction, synaptic plasticity, learning, and memory appear to be partially regulated by AQP4 [44]. In our current results, the reduction in the AQP4/GFAP ratio following sildenafil treatment in ischemic gerbils may provide clues to the improved the LTP effect due to the regulation of extracellular glutamate by glial cells [72,73]. However, the clear mechanism of how the reduction in edema formation due to the attenuation of astrocyte reactivity affects cognitive function requires further research. 

Our results showed differences in the improvement of delayed neuronal cell death in the hippocampus depending on the dosage of sildenafil. However, the protective effect of sildenafil on synaptic structure does not appear to be dose-dependent, but it is effective in improving cognitive behavior. Therefore, our results suggest that sildenafil administration after transient global ischemia may enhance synaptic plasticity, potentially contributing to the improvement of various hippocampus-dependent memory impairments. Further investigation into the changes in synaptic plasticity is needed to clarify these effects. In particular, the NOR test involves various brain regions influencing memory, learning, preference for novelty, and recognition processes associated with the hippocampus and the perirhinal cortex [74,75]. Aversive emotional learning is regulated by neurotransmitters, such as acetylcholine, noradrenaline, and dopamine, in brain regions, including the amygdala, cortex, hippocampus, striatum, and medial prefrontal cortex [54]. Clinical studies revealed that theta oscillations originating from the amygdala temporarily modulate amygdala-hippocampal gamma power coherence, facilitating the encoding of aversive memories [76]. Therefore, our study findings suggest that sildenafil may positively affect other brain regions besides the hippocampus, warranting further investigation.

## 5. Conclusions

The combined results of behavioral and neurobiological assessments support the notion that sildenafil mitigates ischemic damage, particularly highlighted in the attenuation of activated astrocytic responses, as indicated by the double-immunofluorescence analysis of GFAP and AQP-4. The neuroprotective properties of sildenafil in global cerebral ischemia are associated with its ability to regulate intracellular signaling pathways related to memory formation. 

However, certain study limitations must be acknowledged. The study primarily focused on short-term effects, and the long-term consequences of sildenafil treatment remain unclear. The observed dose-dependent response, with the higher dose exhibiting a more pronounced effect, raises questions about the optimal dosage for achieving cognitive benefits. This prompts the need for further exploration into the dose–response relationship and necessitates a nuanced understanding of the pharmacokinetics and pharmacodynamics of sildenafil in the context of cerebral ischemia. The distinctive performance between the pre-treated and post-treated groups introduces a temporal dimension to sildenafil’s cognitive effects. The consistently superior performance of the post-treated groups suggests that the timing of sildenafil administration may be a critical determinant in harnessing its full cognitive benefits. This temporal aspect adds a layer of complexity to the design of therapeutic strategies, emphasizing the need for precise timing considerations in clinical applications. 

Despite these limitations, this research provides a foundation for future investigations into sildenafil’s therapeutic potential for cognitive impairments associated with cerebral ischemia. The multifaceted approach, combining behavioral tests with molecular analyses, contributes to a comprehensive understanding of sildenafil’s impact on post-ischemic cognitive function. Overall, this study opens avenues for further exploration and emphasizes the need for clinical trials to validate these preclinical findings in a translational context.

## Data Availability

The original contributions presented in the study are included in the article, further inquiries can be directed to the corresponding authors.
